# Lived experiences of adults living With HIV/AIDS receiving antiretroviral therapy at werabe comprehensive specialized hospital in Silte zone, Ethiopia

**DOI:** 10.3389/frph.2025.1494390

**Published:** 2025-07-28

**Authors:** Abdulmejid Mustefa, Lonsako Abute, Kemal Lemnuro, Abdulkerim Badgeba, Delwana Bedru, Bisrat Feleke Bubamo, Gizachew Beykaso, Feleke Doyore Agide, Abinet Arega Sadore

**Affiliations:** ^1^ Department of Public Health, College of Medicine and Health Science, Werabe University, Werabe, Ethiopia; ^2^ School of Public Health, College of Medicine and Health Sciences, Wachemo University, Hossana, Ethiopia

**Keywords:** phenomenology, HIV/ADIS, PLWHA, werabe comprehensive specialized hospital, qualitative study

## Abstract

**Background:**

The HIV/AIDS pandemic has had a profound impact on patients’ lives on all fronts, as well as on society. Examining adult PLWHA experiences from various angles helps find gaps, develop plans, and put policies and coping mechanisms in place. Therefore, this study aimed to explore the lived experiences of adult patients receiving ART at Werabe Comprehensive Specialized Hospital in Ethiopia.

**Method:**

A qualitative approach with a phenomenological study design was used from March to June 2022 among 12 purposively selected adults on ART at the Worabe Comprehensive Specialized Hospital. Data were collected using semi-structured, open-ended questions. In-depth interviews were audiotaped, transcribed verbatim, and analyzed using qualitative data management software (ATLAS Ti version 7.1.4). The findings were summarized under five themes by applying inductive thematic analysis.

**Results:**

This study explored five themes that included (I) experiences at the time of diagnosis of their positive HIV Status with two sub-themes: inappropriate counseling and difficulty in accepting positive results; (II) disease management skills with three sub-themes: ART Initiation, Perceived health importance of ART; (III) Enacted stigma with two sub-themes: Stigma & Discrimination and Disclosure; (IV) Experiences towards gaining support with three sub-themes: Family care, Peer/social support from organizations and Hospital and Health professionals’ care; and (V) perceptions towards health status and feelings about their status with three sub-themes of living as normal life, feeling about their HIV status and vision of purposeful life.

**Conclusions:**

Creating educational initiatives and consistently educating the public about health issues can greatly increase public knowledge of HIV, alter perceptions of the virus, and influence how others behave when interacting with people with HIV/AIDS. This mental shift fosters social support and lessens the obstacles to the acceptance of infection by PLWHA.

## Introduction

HIV/AIDS is a global pandemic with cases reported in virtually every country. The goal of ending the HIV epidemic as a threat to public health is fast approaching (Sustainable Development Goal 3.3), so it is imperative to assess current social and behavioral trends and track global progress towards reducing HIV incidence and mortality (1). In 2022, 39.0 million people were living with HIV. Women and girls made up 37.5 million adults (15 years and older) and 53% of all HIV-positive individuals. Approximately 86% of people living with HIV were aware of their HIV status; however, approximately 5.5 million people were unaware that they were HIV positive (2, 3). Following that, 2022, 29.8 million people had access to ART, up from 7.7 million in 2010. In 2022, 76% of people living with HIV received treatment. Approximately 77% of adults aged 15 and older living with HIV had access to treatment (3).

With significant advances in antiretroviral therapy (ART), AIDS has progressed from acute to manageable chronic (HIV) infection, with improved immunity, viral suppression, and health-related quality of life in people living with HIV/AIDS in various countries, including Uganda, Tanzania and Ethiopia (1, 4–8). This progression has changed the lived experiences of PLWHA, exposing them to learning, coping, and managing their condition (2, 9). Ethiopia has a clear policy to improve access to medication and the quality of life of people living with HIV, and the national ART coverage has increased. However, living with HIV remains challenging for multiple complex reasons, including socio-demographic, economic, physiological, and psycho-emotional disturbances that have occurred in the lives of individuals living with HIV/AIDS (10).

The phrase lived experience pertains to the natural world in which humans live or exist through and respond to experiences. HIV/AIDS refers to the content of thoughts, feelings, and observations in the context of adults living with HIV, starting from the date of a positive diagnosis, undertaking ART and self-management skills, and the overall quality of life and feelings towards HIV/AIDS (11).

Hence, exploring the lived experiences of adult PLWHA is important to institute appropriate interventions to minimize burdens related to negative experiences, to enhance positive experiences for adhering to their treatment, self-care, and improving quality of life, and to prevent mortality and morbidity related to HIV/AIDS and its complications. Thus, this study aimed to explore the lived experiences of Adults who are on ART to properly address issues related to adult PLWHA.

Following significant advances in antiretroviral therapy, there has been an improvement in the quality of life of PLWHA owing to the progression of the disease from acute to chronic manageable. However, living with HIV remains a challenging condition for multiple and complex reasons, including lack of knowledge/awareness about the disease, lack of access to testing for vulnerable groups, poverty, low educational literacy rate, inadequate knowledge of healthcare professionals, stigmatizing attitudes toward patients, bad feelings at the time of positive diagnosis, delayed initiation and non-compliance to ART, stigma and discrimination and its consequences, lack of hope in their health status, and lack of vision for their future life (1–3).

Individuals living with HIV/AIDS started notifying and living with positive and negative experiences from the time of a positive diagnosis of the disease. For instance, the literature highlighted that healthcare professionals' improper way of informing the diagnosis failed to protect privacy and confidentiality, and discriminative behaviors towards PLWHA (1, 2).

Another aspect of the lived experience of PLWHA following a positive diagnosis of the disease is the initiation of ART and self-care. Most PLWHA patients have a reduced willingness to seek medication attention, difficulties in initiating ART, non-compliance with ART, and poor self-management skills (10–12). The reasons for delayed initiation of ART outlined in the literature include the following: denial of status (10, 13, 14) fear of side effects (11) spousal influence (15), poverty (16) religion (17) initiation protocol (18) and shortages of staff (19) as well discrimination and HIV-related stigma (20). Adherence to ART is a dynamically complex behavior influenced by socioeconomic and cultural factors (21). Specifically, forgetfulness, fatigue, hopelessness (22) stigma and discrimination (23), IV non-disclosure (24) and religious beliefs, adverse drug reactions, lack of social support, alternative therapies, COVID-19-related lockdown, and fear of less COVID-19 care due to HIV-associated stigma were identified as barriers affecting compliance with HIV care & ART (22). On the other hand, family support, access to affordable transportation (25) positive relationship with healthcare providers (26), livelihood support, and attending a support group (27) improved knowledge about the disease (28), use of reminders (26), carrying ART while away from home (29) and initiation of telephone consultations, courier delivery, and long-term delivery of antiretroviral drugs during COVID-19 were identified as facilitators of HIV retention (24). Besides the suffering caused by the disease, PLWHA faces other challenges to overcome, including stigmatization, discrimination, and the issue of disclosure. Through stigmatization, PLWHA often becomes an object of scorn, hatred, violence, and death (30). HIV/AIDS status disclosure has positive and supportive responses, behavioral changes, and public health benefits, such as HIV prevention advocacy, HIV testing, protection from infection, and early enrolment in ART (31).

Despite studies showing that the utilization of ART services from previous studies focuses merely on quantification of the practice rather than ART experiences, and different struggles are taken from the government and researchers' side to improve their quality of life, to the investigators' knowledge, no studies have been conducted in the study area on the experience of Adults Living With HIV/AIDS and On Antiretroviral Therapy. This study fills this gap by exploring the experience of adults living with HIV/AIDS and on Antiretroviral Therapy in Werabe Comprehensive Specialized Hospital, Silte zone, southern Ethiopia.

## Materials and methods

### Study area and period

This research was conducted at Werabe Comprehensive Specialized Hospital in the Silte Zone, South Ethiopia, from May 1 to June 30, 2022. The hospital is located in the outlying town of Werabe, which is the administrative center of the Silt Zone, approximately 173 km south of Addis Ababa. As evidenced by the hospital, it is a referral and comprehensive specialized public hospital in the country, trying to fulfil the specialized health needs of the people through the provision of preventive, curative, palliative, and rehabilitative services in collaboration with stakeholder groups for more than five million people from the southern and central parts of Ethiopia, including the Silte and neighbouring zones (Halaba, Gurage, Hadiya, and Kembata) and region (Oromo). With a bed size of 600, the WCSH provides services for approximately 10,000 inpatients, 160,000 outpatient attendants, 11,000 emergency cases, and more than 4,500 deliveries per year. It also serves as a teaching hospital for several undergraduate and postgraduate programs in the field of basic sciences as well as clinical medicine for health science students at Werabe University and St. Paul Millennium Medical College. The hospital has many follow-up clinics for both adult and paediatric patients, of which the ART follow-up clinic is one of many chronic follow-up clinics. The WCSH 2014 EFY Annual Activity Report states that there were approximately 51 deaths at WCSH 189, 1st line 180, Adult 141 Pediatrics, 9 2nd line 33, Versus 2 Deaths, 4 at Zonal 1,342 (Adult 1,257 Paediatrics), and approximately at Zonal 1,342 (Adult 1,257 Paediatrics). The service is rendered by physicians and nurses, and runs on all working days of the week.

### Study design

A descriptive phenomenological study design was used to explore the experiences of adults living with HIV/AIDS on ART.

### Populations

Study population: All adult PLWHA received antiretroviral therapy and were followed up at the Worabe Comprehensive Specialized Hospital during the data collection period.

### Recruitment of the study participants

**Figure 1:**
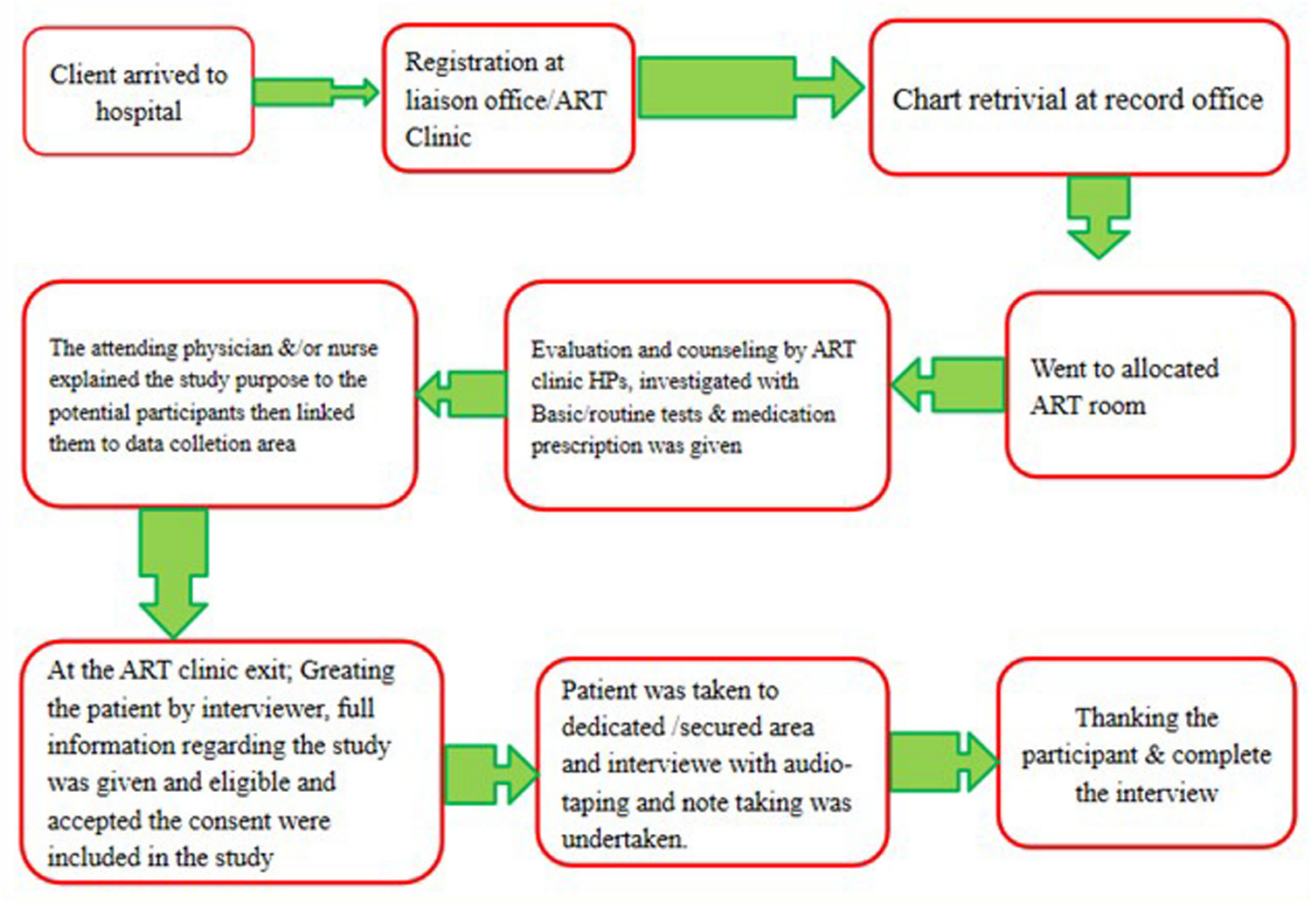
Flow diagram of study participant's movement through ART clinic, recruitment and data collection process.

A Purposive sampling was used to recruit study participants. Twelve adults on ART were selected for the interviews. The number of participants was determined based on the saturation level of information. Saturation occurs when more participants are included in the research, which does not result in obtaining additional information. By bracketing ourselves, we used probing in the interviews to obtain sufficient information on the study. The data were collected until no new or relevant information was obtained (Figure 1).

### Data collection

In-depth audio-recorded interviews were conducted using a semi-structured interview guide developed by reviewing related literature and guidelines considering the context of the study (3, 26). Interviews were conducted in a secure area (private room) at the ART clinic. After obtaining informed consent, the principal investigator conducted all interviews to ensure consistency in data collection. Prior to the interviews, the attending physician and nurses explained the purpose of the study to the potential participants and then referred them to the (private) room. Field notes were simultaneously incorporated into transcriptions. The recruitment of participants was continued until no new data were found (until data saturation). The point of saturation was determined by repetition or redundancy of information. The unit of the enquiry was individual patient following ART. The length of the interviews ranged from 35 to 55 min. No new information was obtained at the 12th interview; hence, the saturation point was believed to have been achieved at this point.

### Trustworthiness

Conformability was ensured by keeping the details of data collection, analysis, and interpretation of the data checked and re-checked throughout the data collection and analysis. The data were documented using a clear coding plan that identified the codes and explained the themes identified in the analyses. To ensure dependability, the findings of the chosen methodology, selection and recruitment of participants, data collection methods, and analysis process are described. A detailed chronology of research activities and processes, data collection and analysis, emerging themes, categories, or quotations was audited by advisors and colleagues and examined by another person who had experience in conducting qualitative research to assure dependability.

To ensure credibility, the investigator and data collector set aside personal experiences and presumptions to illustrate the true picture of the participants' accounts (bracketing mind). The investigator and data collector took adequate time with the participants, and the study participants were approached without pressure and interviewed in a comfortable environment. The interviews were semi-structured and open-ended, and the respondents were allowed to discuss the questions in an uninhibited manner while being guided to remain focused on the topic of interest. Appropriate probes were used to obtain detailed information about the responses. Some of the transcripts were coded by a colleague and compared for similarity, and the analysis was examined. Member checking was performed at the time of the conversation, and at the end of each data collection, the participants were given a summary of what they had said in order for them to confirm that it was what they wanted to say. To ensure transferability, a thick description of all the inquiry processes and findings was made so that any reader of the report would be able to use and researchers could replicate the study in other settings that have similar conditions.

### Data analysis

The recorded materials were transcribed verbatim in the local language and then translated into English by the researchers and research assistants. Field notes were simultaneously incorporated into transcriptions. Coding was performed by reading and re-reading the compiled transcripts using ATLAS qualitative data management software. ti version 7.1.4. Before the actual coding began, the transcripts were independently read by the investigator and the research assistant, and then a codebook was developed. The codes were organized to create categories and themes. The data were analyzed using a thematic analysis. Finally, the results are presented using the major themes and categories supported by quotes to describe the overall essence of the experience. An inductive approach was implemented to identify themes and categories.

## Results

### Socio-demographic characteristics

Twelve (eight male and four female) HIV-infected adults, aged 29–51 years, with an ART duration of 7–21 years, participated in the study (Table 1).

**Table 1:** Socio-demographic characteristics of study participants (of adult PLWHA and on ART) in werabe comprehensive specialized hospital, 2022.

Participants	Age	Sex	Marital status	Occupation	Educational level	Duration on ART	Residency
P-1	32	F	Divorced	Cleaner	Diploma	14 year	Werabe
P-2	38	M	Married	Farmer	Illiterate	8 year	Alicho
P-3	46	M	Married	Merchant	Grade 8	14 year	Werabe
P-4	32	F	Widowed	Merchant	Grade 10	12 year	Dalocha
P-5	33	F	Married	House wife	Diploma	11 year	Butajera
P-6	43	M	Married	Civil Servant	Diploma	21 year	Werabe
P-7	37	M	Married	Driver	Degree	9 year	Werabe
P-8	51	M	Divorced	Farmer	Grade 4	12 year	Alkaso
P-9	29	F	Married	Civil Servant	Degree	4 year	Werabe
P-10	29	M	Single	Civil Servant	Degree	6 year	Sankura
P-11	33	M	Widower	Merchant	Diploma	7 year	Dalocha
P-12	39	M	Divorced	Farmer	Grade 8	12 year	Dalocha

Key: ART, antiretroviral therapy clinic.

### Overview of participants' lived experiences

Analysis of the lived experiences of adult PLWHA and ART at WCSH by descriptive phenomenology identified five main themes and 13 sub-themes, as shown in Table 2, which will be described subsequently (Table 2).

**Table 2:** Main themes and Sub-themes of the study (of adult PLWHA on ART) in worabe comprehensive specialized hospital, 2022 .

S№	Themes	Sub-themes
1	Experiences at time of diagnosis of their positive HIV Status	Inappropriate Counseling
		Difficulty in accepting positive result
2	Disease management skill	ART Initiation
		Perceived health importance of ART
		Self-care
3	Enacted stigma	Stigma & Discrimination
		Disclosure
4	Source of support	Family care
		Peer/social support from organizations
		Hospital & Health professionals’ care
5	Perceived health status and feeling about their HIV/AIDS status	Living as normal life
		Feeling about their HIV status
		Vision of purposeful life plan

Key: ART, anti-retro-viral therapy.

### Theme I: experiences at time of positive diagnosis of HIV

#### Sub-theme 1.1: inappropriate counseling

The results highlighted that participants perceived that healthcare professionals did not properly inform them about the diagnosis, failed to protect patients' confidentiality, and exhibited discriminative behaviors towards them during their positive diagnosis.

The study participants stated that they were tested for HIV without knowledge when they visited a health facility for other services. Moreover, they asserted that they were not informed about the preliminary test results when asked to provide a blood sample for confirmation/verification; thus, they felt that healthcare professionals withheld information from them. A 33 year old women participant stated the following.

“ … I was 5 months pregnant by the time I heard my positive test result …… I have given sample for routine investigation and they did not informed me as they prescribed test for HIV … They need to redraw blood from me and I asked why? They said they couldn’t tell it at that moment. It was exactly like that. I said “You have to tell me because you requested me to come again. You want to draw blood, it's my right to know” “No, we don’t want to disturb you by saying it now”. That's exactly what they said …… ” (P-05)

#### Health professionals prejudiced towards infected or discrimination

Participants also stated that certain behaviors on the part of healthcare professionals caused them to feel humiliated. A 37 year old male participant said,

“You sit down there”, she said to me. She opened the windows and doors, moved away from the desk, and took a piece of paper. It was only 15 min after I received the diagnosis. “How old are you? She asked me directly. Then, she asked if I drank alcohol, smoked, had a nightlife, had had a lot of partners in the past six months … Well, at first, I had a humming noise in my head, but then it faded, and it turned to curiosity; I wondered what this woman's intention was. (…) I remember her reopening of the window when it was closed a couple of times. (…). I then left the room and torn the test results into pieces”. (P-07).

#### Sub-theme 1.2: difficulty in accepting positive results

Patients experience an unpleasant phenomenon in the face of a positive diagnosis of the disease, which manifests as an escape from reality (denial), feelings of guilt and regret, motivation for social isolation, fear of disgrace, and suicidal ideation.

#### Denial of status

The lived experience of these patients shows that after receiving a positive diagnosis of the disease, they found that this fact was difficult to accept and believed that the test result was wrong or that the result belonged to someone else. For this reason, the patients referred to other laboratories after receiving the first positive diagnosis of the disease A 43 year old male participant said that:

“ … After hearing the lab result and found out what the disease really was, I was really shocked and said it was impossible, it was definitely wrong and it's not true … I could not believe it at all, because I had no exposure like multiple sexual partner and this could not happen to me …… I went to Butajira hospital and there I was again tested for HIV and … ” (P6).

#### Guilt and regret

The experience of these patients has shown that after receiving a positive diagnosis of the disease, they consider themselves guilty, complain about themselves, and feel regretted by their past lifestyles. These patients condemn their lifestyle, sometimes even consider themselves as deserving of the disease, and think that it is a ransom that they have paid back. A 43 year old male participant said,

“ … after getting the disease, I realized that I was paying the ransom because I was hundred percent guilty, I was the one who caused this situation with a series of bad deeds, and now I have to be punished … ” (P6).

The other patients stated that he felt remorseful for his lifestyle and actions as soon as he heard a positive diagnosis of the disease because he thought that if he had lived a healthier lifestyle, he would not have been infected by stating A 33 a old male participant said that:

“ … After realizing may status, I was very sorry for my past, because I really did not have a healthy life. I made a series of mistakes that caused me to get caught. At that moment, I just regretted why I had this disaster … ” (P11).

#### Feeling social isolation

The experiences of these patients showed that after facing the incident, they suffered an internal failure that caused them to try to distance themselves from others. These patients have become isolated, withdrawing from the community and sometimes even from their families. A 37 year old male participant said,

“ … After this incident, I decided to live alone forever and stay away from all my family members even changed my residence. I made a good excuse and broke up my marriage and changed my residence from Harar to Werabe town and started living alone in rent house …… ” (P7)

#### Fear of disgrace

One of the feelings that these patients experienced when faced with a positive diagnosis of the disease was fear of disgrace. They suffer from the perception that the spread of news about the illness hurts the attitudes of those around them, causing them and their families to be discredited. A 38 year old male participant said,

“ … My family was well known and reputed in our village and I was also active participant in our family and community issues so It was very annoying for me when I thought I would no longer be active participant and I felt I and my family no longer have a reputation and everyone would think badly of me and my family … ” (P2).

#### Suicidal ideation

The patients stated that they were so upset with the positive diagnosis of the illness and they immediately thought they could not live with the fact and the best thing to do was to end their own lives A 39 year old male participant said that:

. “ … The news was so bad for me that I immediately thought that if the test result was correct and I had AIDS, I would have to kill myself and end this wretch life, oh, I had a lot of problem and the thought of having to wait for a gradual death was horrible to me … ” (P12).

### Theme II: disease management skill

#### Sub-theme 2.1: about ART initiation

As emerged from the data analysis, the reasons for delayed ART initiation among the study participants included denial of status, fear of being exposed, High CD4 count, and fear of the side effects of ART.

#### Perceived stigma

The lived experience of these patients showed that initiating ART means exposing themselves to their family and friends; therefore, the patients preferred not to take ART to hide their positive status. A 29 year old male participant said that: as follows:

“ …… Since I was living with my family by the time I was told to be positive I have nowhere to hide the drugs, so I decided not to take ART until things become adjusted .… ” [P10].

Stigma and discrimination is common once it is disclosed within a family or friends. ART users including me need more privacy in using drugs and treatment. However, nowadays, people are aware of the diseases and stigma is decreasing.

#### Decision by health care provider

The study revealed that a high CD4 count hinders the early initiation of antiretroviral therapy. A 33 year old women participant remarked,

“ …… I heard my positive HIV status while I was sent for routine lab test for Antenatal care follow up and health care professionals working at Antenatal care clinic informed me to wait for CD4 count to decide initiating ART and by that time, my CD4 was 950. They said it was good and told me to have follow up checkup of CD4 count every six months …… ” [P5]

N.B. Reason: Test & Treat approach was not implemented by that time.

#### Fear of side effects

This study revealed that anticipation of drug side effects would hinder the early initiation of antiretroviral therapy. A 32 year old women participant follows: “ *…*… *You know I saw how the drugs did to my husband, so when I see myself I was healthy, I could work like before and I did not want to face the treatment side effects.…”* (P1).

#### Sub-theme 2.2: perceived health importance of ART

The participants understood why they were receiving treatment, the magnitude of missing antiretroviral therapy, and they believed that the medicine worked well and that they could live as long as HIV-negative people live, and the participant obeyed and took the medication because of knowing the consequence of not taking the medication.

A 38 years old male participant stated that “ …… *If I don't come my home and take my medicine on time to stay alive and well, who will feed me when I get sick tomorrow? So I have to take my medication regularly on time to have healthier life* *….* *”.* (p02).

This idea was shared by A 43 years old men participant “ …. *I see taking medication properly as my life because I take proper care of myself, my wife, my children, and my family* *….* ”. (P06).

Also A32 old female participant stated that “ *…* *just as I do not sleep without eating food, I do not sleep without taking it, ‘ I carry my medicine with me wherever I go; I didn’t skipped an hour, because it is my life*”. (P01).

A 32 years old women participant stated that “ …. *After I started the medication I am healthy, Alhamdulillah* *…* *previously, I stopped taking the medication for few days, and I'm exposed to Breaking of the lips, Lip wounding and my nail was removed. I mean that it helps me I have been to be very cautious to take the medication. I think that it also Protects from the co-morbid disease and will help me stay down the viral load”.* (P04).

#### Sub-theme 2.3: self-care and disease management skills

In this study, the participants who felt healthier after taking medicine regularly became convinced that the medicine they took had good benefits for them and helped to live with the loved ones children, wives, or parents in terms of wanting to be happy and wanting to see their children grow up well. A 39 year old male participant said “*I always eat before taking medicine so I don't feel sick; I immediately go to clinic if I feel abnormal”. (P12).*

A 33 year's old male participant stated that “ …. *I just talk to my family about the disease; I eat and drink appropriately because I want to have good nutritional status to prevent from opportunistic infection*”. (P11).

Additionally, a 32 year's female participant said that “ *…* *I have become myself; I became aware of my health and used it for myself, and then I began to counsel people and my body returned to normal. No matter what time it is, I will take my medication on time*” (P4).

Also, a year 37 years old male participant state *“I have three child and they need my help* *…* *so I have to be healthy and work hard to get money and*  *…*  *I want to see my child grow up and got good work opportunity* *…*  ”(P7).

#### Theme III: enacted stigma

This study highlights the challenges that adult HIV/AIDS patients encounter in their daily lives and their interactions with family, friends, and community members. The following categories were recognized: (1) Stigma and discrimination and (2) Status Disclosure.

#### Sub-theme 3.1: stigma & discrimination

Participants believed that HIV patients experienced discrimination from both individuals and society, which negatively affected their career aptitudes. Due to the unfavorable cultural perspective of HIV, they face some type of stigma, such as social marginalization and community prejudice, even from their family members and neighbors.

A 43-year-old male study participant stated that *“* *…* *even though the rate has reduced a lot, it is not fair to say that there is no embarrassment. Because even so-called literates and individuals, even my colleagues, ask silly questions like “does he* have *HIV/AIDS?” when they see me with someone who accompanies me on my way. Because of this, even my close friends were isolated from me in our day-to-day activities like going to the work place, market, and so on*”. (P06).

Additionally, a 29-year-old male study participant mentioned being marginalized by stating, “*In different public areas like marketplaces, I have seen and heard people pointing fingers towards me and saying to each other that that girl has HIV/AIDS, and while I am trying to approach them, some of them go away*” *(*P10).

Due to community stigmatization, many of the study participants reported that they incurred additional societal and economic burdens, such as being exposed to transportation costs to receive HIV treatment from health facilities further away from where they lived, even though there was an HIV care facility available in their local community. This was because they did not want to be treated in their local health facilities because of the fear of stigma and discrimination. This creates an additional financial burden and is a potential risk factor for poor social interactions.

A 38-year-old male participant testified as follows: “*Though there is an HIV care clinic in the nearby health center and there is difficulty in accessing transport, I choose to have follow up at here, WCSH. because I am free from those negative people who are trying to prejudice me and by doing so I got relief and free from negative thoughts*” (P02).

Another participant isolated themselves from the community because of their physical appearance and negative attitude toward their illness, which pushed them to think more about the diseases, feel rejected and ignored, have low self-esteem and low self-efficacy, and finally isolate them. This was a common experience among participants.

A 32 years old female study participant said that: “ *…* *Yeah, I had isolated myself from the community, even decided to change my name; and since I was so thin I used to run away from people because of thinking about how I came to be like this*”. (P01).

A 39 year old male study participant also stated that: “*I don’t know why, when people saw me in public place I feel like I am different from them and feel shame, this bad perception made me isolating myself from the community*”*.* (P12).

#### Sub-theme 3.2: disclosure

Maintaining secrecy or limiting disclosure of HIV status appeared to be a protective strategy among many participants. A 38 years old male study participant also stated that “*I take my drug in a hiding place when my child sleeps since she doesn’t know about my status. I always come from a remote area which takes 2 h on-foot and by bus since there is still a negative attitude towards the disease in our community. If I get service in my locality, my status could be exposed and my social life and work could be affected* *……* ” (P-02).

Additionally, A 29-year-old female study participant stated, “ *……* *A few years ago, while we were at a community gathering (mourn) of an individual with HIV/AIDS, they (individuals) started talking about the situation and started to blame him and said who dared to bury his corpse! This incident reminded me that the community cannot accept and have a negative attitude toward HIV-positive people. This is why I would never talk about my status* *……* *”* (P-09).

*The above idea was shared by a 37-year-old male participant who stated that: “* *….* *I did not want anyone to know my HIV status because If others know they will not support me, rather they will blame me and space themselves away, so I chose not to disclose my status”.* (P-07).

This idea was also shared by another 51-year-old male participant: “ ….  *I did not tell anyone except a few of my family because I fear the perception and public undesirable views*”*.* (P-08).

In this study adult, adults with PLWHA experienced varying degrees of stress because they either hidden the illness from their families or asked family members to assist in concealing it. Therefore, adult PLWHA should disclose the illness to their families and friends to decrease stress. The stress resulting from recurring back-and-forth consideration of HIV status disclosure often creates apprehension and restlessness. The participants described this experience as follows:

A 32-year-old female participant said that “*I hide the anti-retro viral medication in a secured area (cabinet) and I do not take it in front of anyone, even my family. Once upon a time, I told my brother to bring the money from a cabinet where both the money and the medicine were put together, and when I remembered the situation, I experienced involuntary urination and a headache due to fear of being exposed by my brother*”*.* (P-04).

Participants claimed that if they disclosed their status, others would criticize and judge them, so they preferred that their treating medical professionals be aware of it. They consider who their helpers are, and most prefer to disclose their status to their close relatives, especially their children.

A 43-year-old male participant stated, “*You have to tell the person who you think is good and going to take care of you and take care of my children. And by exposing yourself, you will reduce a lot of your inner pain*” (P06).

A 29-year-old female participant also stated*, “…If you don’t know a person who is on your side or with whom you have an intimate relationship, you didn’t tell your status to anyone. That's why I told my best friend first”. (P09).*

#### Theme IV: lived experiences of adult PLWHA support status

The following sub-themes were included in the main theme: support from family, support from peers, and assistance from medical professionals.

#### *Sub-theme 4.1*: family support

Participants received support from their families in the form of instrumental support, which was accompanied by assistance in taking drugs on a regular basis. The family provides them with informational support by reminding them when they take medication.

A 43-year-old male participant stated that “*My family, especially my eldest son, reminds me that Daddy, it is time to take medication. Because I told my children that I couldn't live without the medicine”*. (P06).

The above idea was also shared by a 32-year-old female study participant*:*“ *…* *my children know more about the medicine and they try to remember me; they know the time, they bring it and put it next to me in order to take the medication*”. (P01).

The above idea was also shared by a 32-year-old female study participant*:*“ *…* *my family helps remind me, to take my medications regularly and even to carry medicine wherever I go*” *(P06).*

#### Sub-theme 4.2: peer support (from organizations)

Their fellow PLWHA members in Werabe Town have an association called MAMANI in which they remember one another when sharing drugs.

A 33-year-old male participant stated, “ *…* *I have close friends (with PLWHA) in our organization called MAMANI at Werabe and once I forgot my follow-up schedule due to another social problem (mourn) and my friend called and reminded me to come and take my medications*”. (P11).

A 33-year-old female study participant shared this idea, “*I get a solution from my friends to solve my problem many times* *…* *I am reminded by my friend to take medicine and when I get sick they take me to the clinic*” (P05).

#### Sub-theme 4.3: support from hospital and care providers

Nearly all participants in this study had extensive follow-up and hospital engagement, so they were fully aware of their surroundings. They are friendly to the clinical staff, including doctors, nurses, and other employees. A minority of the participants reported positive interactions with, support from, and encouragement from clinical services.

A 32-year-old female participant stated that “*I'm always happy to talk to the health professionals because they (health professionals) help me by advising about the medication, the disease, and the lifestyle. Thank you for them*”. (P01).

A 32-year-old female participant shared this idea*: “* *…* *healthcare professionals gave me medication that was taken three times a day, and they told me that it should not be stopped. I have a good relationship with them. I got plenty of help from them*”. (P04).

Participants in the study stated that doctors and other healthcare workers at the hospital's ART clinic gave them friendly treatment and expert advice about their future lives, and directed them to NGOs working in the area.

A 33-year-old female participant stated that:“ *…* *Look, he said, “we are here with you whenever you have a medical problem.” He handed me a piece of paper in which there was information about the foundation. He said, “You can contact them; they have a good counselling service. You can find any information you need there”. Then I received relevant information from them*”*. (P-05).*

#### Theme V: reported health status and lessons learned from adult PLWHA about how they feel about their HIV/AIDS status

This theme contained a description of what the participants perceived about their health and feelings about their HIV/AIDS status. It has three sub-themes: (1) living a normal life, (2) feelings about HIV status, and (3) future life plans.

#### Sub-theme 5.1: thought of living as a normal life

The participants explained that they took it easy when they heard their status first, and they also reported that they were healthy and confident enough to perform as anyone else would.

A 32-year-old female participant stated, “ *…* *It doesn't matter; I can live like any other person lives. I feel healthy and I think I can perform anything that can be performed as that of any healthy person* *….* *” (P01).*

Another 32 years old female study participant said “*Living with HIV/AIDS is nothing, it does not matter if you give up what the disease didn’t need to and doing what the disease want to do*”. (P04).

#### Sub-theme 5.2: feeling about their HIV status

Those who express such feelings have also been reported to experience agitation/stress and frustration. However, over time, they forget that feeling and believe they are healthy and stress free. A 32-year-old female study participant stated this situation as follows: “ *……* *Everyone become shocked and confused at that moment when they hear his/her positive status. The same is true for me. Just I feel some terrifying thing has occupied my whole body but if you think well without confusion and return to yourself, it is easy. Accepting HIV positive status as a coping mechanism* *….* ”(P04).

Another 46 years old male study participant described that HIV/ADIS by itself cannot harm and believed that it is nothing by saying that: “*……* *I told myself, I did not have HIV/AIDS. I didn’t believe that the disease itself does not cause any harm, so I still feel like I don’t have this disease in the future”. (P03).*

#### Sub-theme 5.3: adult people's living with HIV/AIDS life plan

They wanted to demonstrate that they were capable of doing everything. Some dreams of becoming well-known and active participants in their communities. A 32-year-old female participant stated that: described her wish as follows: “ *……* *I have a dream to make pilgrimage to mecca (for haj) and also to become reputed elder who settles disputes arising in our community* *….* ”(P04).

**Figure 2:**
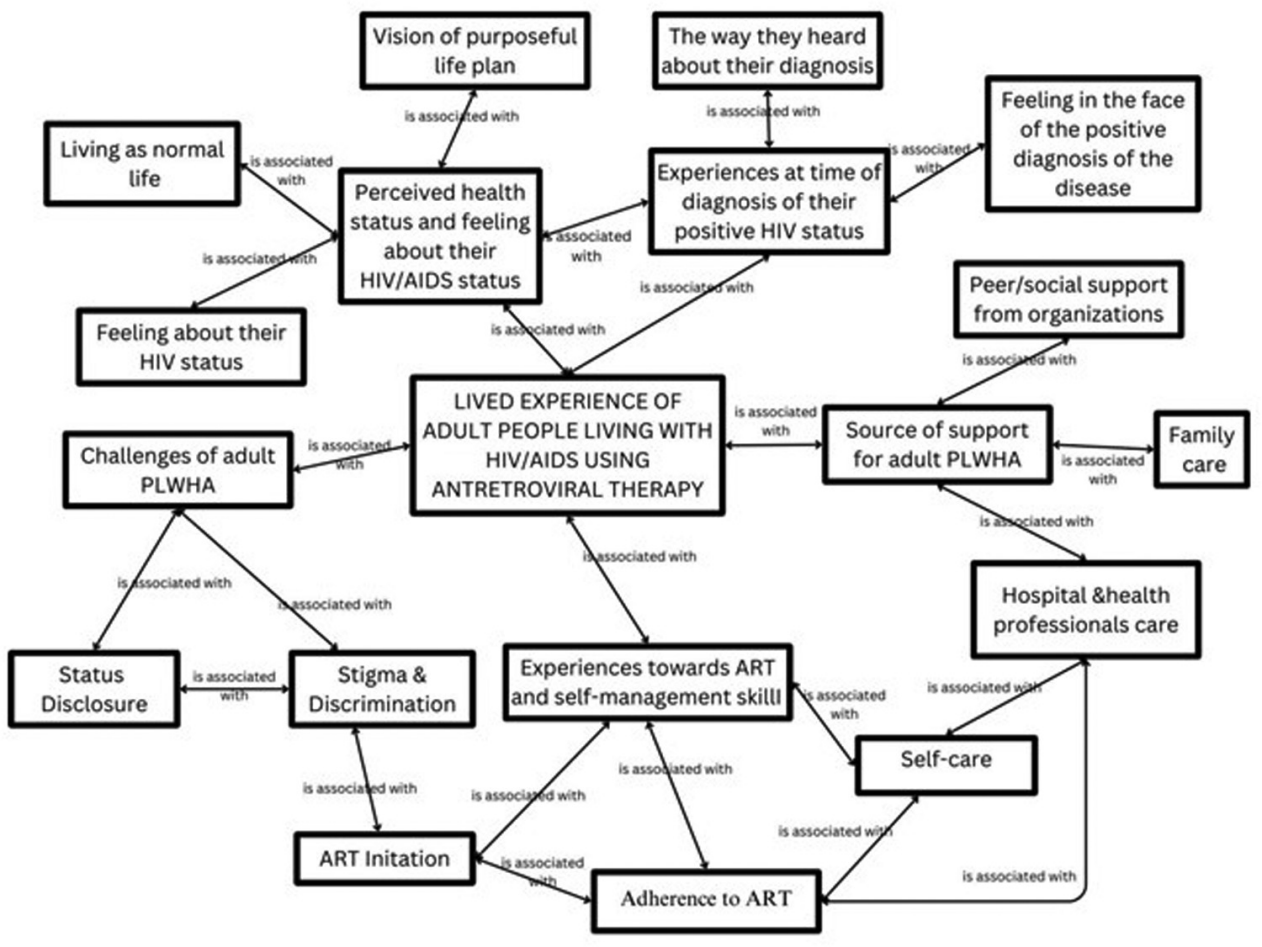
Concept coding.

Another a 29-year-old female participant stated that: discussed his wish as follows: “*I want to be counselling psychologist; I want to work on forgotten people*. *You know when you close and talk them; you get what you didn’t expect*. *They want to share their ideas but they don’t get the one who hear them*. *So I will be happy if I reach them and give them what can I do” (P-10)* (Figure 2).

## Discussion

This phenomenological study used a descriptive qualitative approach to investigate the positive and/or negative lived experiences of adult HIV-infected individuals in various aspects of their lives, with a focus on challenging experiences. Based on the data analysis, five main themes and thirteen sub-themes emerged regarding the experiences of adult HIV-infected individuals: (1) experiences at the time of diagnosis of their positive HIV Status, (2) disease management skills, (3) enacted stigma, (4) Source of Support, and (5) perceptions towards health status and feelings about their HIV/AIDS status.

These results indicate that confidentiality and privacy are not respected when individuals are realized. In addition, people felt guilty and afraid of the sickness as a result of the medical establishments' reaction to bad laboratory results. This result is in line with that of other studies done in Turkey (32). They found that healthcare professionals did not inform the diagnosis properly, failed to protect the patient's confidentiality, and exhibited discriminative behaviors towards them, which is also similar to a study that identified healthcare professionals as prejudiced regarding HIV-related issues and lacking accurate/adequate knowledge of the disease (2). This might be due to poor knowledge about the nature of the disease and a lack of appropriate training and approaches while revealing positive test results.

In addition, the confrontation of these patients with a positive diagnosis of the disease caused them to experience bad feelings manifested by status denial to escape from reality, feelings of guilt and regret, motivation for social isolation, fear of disgrace, and suicidal ideation. These findings are similar to those of a study conducted in Hamadan, Iran (7) and similarly, where HIV-infected patients who have recently been diagnosed with the disease experienced anxiety and depression (32) due to social isolation (33–37), regret for the past (7, 38) loss of self-esteem, and severe stress have led to high suicide rates (39).

Another experience of adult HIV-infected individuals is related to ART initiation, adherence, and self-care. In this study, most participants experienced delays in initiating ART due to denial of status, fear of exposure, High CD4 count, and fear of the side effects of ART. In line with these findings, various studies have shown similar findings as a reason for delayed initiation as denial of HIV-positive sero-status (13, 14, 36), fear of side effects of the treatment (11) and initiation protocol concerning high CD4 count (>350 cells/mm3) that patients might experience before the implementation of the national test and Treat strategy (18). Contrary to the findings of other studies, staff shortages were not raised by our study participants as a barrier to delayed ART initiation. This might be because there was enough trained staff at the ART clinic of the hospital.

With respect to ART adherence in this study, most of these participants understood the importance of taking care of their health by adhering to the treatment As identified as facilitators of HIV retention, these factors include having family obligations, wanting to be healthy, understanding the good effects of ART, having supportive friends and family, carrying ART while away from home, and using reminders. This finding is consistent with the findings of various studies, such as family support (36), positive relationship with healthcare providers (28) use of reminders (26) and carrying ART while away from home (29).

On the other hand, stigma and discrimination appeared to be the most challenging and concerning issues for PLWHA as barriers to ART adherence and maintenance of care; similar findings were reported in previous studies in Ethiopia (35), Sub-Saharan Africa (40), America (41), Middle East (42), Asia (43) and Europe (44), all of which found stigma to be a significant contributor to non-adherence. However, the disclosure of a positive status to others is another major concern in adult PLWHA. Fear of isolation, loneliness, stigmatization, and discrimination were barriers to status disclosure. This finding is similar to those of various studies (31, 45, 46).

Families and caregivers were the major sources of support for adult PLWHA. Similar findings were observed in other studies, indicating that family care is a significant source of support for adult PLWHA (35, 47). Nearly all study participants cited healthcare services and service providers' approach as additional significant sources of support, which is similar to research conducted in Turkey (32). This may be because medical personnel treat patients with respect to their care. This study discovered that supporting those living with HIV/AIDS involved belonging to local groups and developing close relationships with their peers.

This study showed that adults living with HIV/AIDS can lead normal lives without the virus because of their desire to continually maintain their health to realize their vision; this is accomplished (by having a purposeful life). These results are consistent with research conducted in Ethiopia (35). The participants stated that they enjoyed thinking about their HIV status, and several also stated that they experienced agitation and loneliness when they did. Those who expressed these emotions also said they experienced agitation, stress, and annoyance. These results are consistent with those of numerous other studies (4, 5, 48).

## Conclusions

In conclusion, this study explored adult HIV-infected people's challenges from the moment they receive a positive diagnosis, throughout the initiation and continuum of care, and negative perceptions of their health and the disease, indicating that they require smoother approaches and more emotional and informational support when they receive a positive diagnosis. A coordinated multidisciplinary approach involving various stakeholders that provides information, counseling, awareness, and facilitates interaction between PLWHA and the community, tailoring societal education for behavioral changes and social communication, and minimizing negative perceptions of PLWHA is critical to reducing stigma and discrimination. Creating dialogues and behavioral exercises to encourage HIV disclosure and increasing support/care are required for early ART initiation, adherence, and continuum of care, which is an effective way to improve PLWHA clinical outcomes and quality of life while also lowering morbidity and mortality from HIV/AIDS and its complications.

## Data Availability

The raw data supporting the conclusions of this article will be made available by the authors, without undue reservation.

## References

[1] Senyurek G Kavas MV Ulman YI. Lived experiences of people living with HIV: a descriptive qualitative analysis of their perceptions of themselves, their social spheres, healthcare professionals and the challenges they face daily. BMC Public Health. (2021) 21(1):904. 10.1186/s12889-021-10881-y 33980195 PMC8117647

[2] Bayrak B Keten S Fincanci M. Saglik Çalisanlarinin HIV/AIDS olgularina yaklasimlari/attitude of health personnel towards people living with HIV. KLIMIK Dergisi. (2014) 27(3):103. 10.5152/kd.2014.30

[3] World Health Organization. The Advanced HIV Disease Research Landscape. Geneva: World Health Organization (2024).

[4] Ddungu G. An Exploration of the Lived Experiences of Individuals Living With HIV/AIDS in Rural Uganda (Doctoral Dissertation). Minneapolis, MN: Walden University (2023).

[5] Philip AA King J Durham J. Lived experiences of persons with disabilities living with HIV in accessing HIV services in Africa: a qualitative systematic review. Disabil Rehabil. (2023) 45(6):937–49. 10.1080/09638288.2022.2051079 35298321

[6] Mhode M Nyamhanga T. Experiences and impact of stigma and discrimination among people on antiretroviral therapy in Dar es Salaam: a qualitative perspective. AIDS Res Treat. (2016) 2016(1):7925052. 10.1155/2016/7925052 27110395 PMC4823479

[7] Imani B Zandi S Khazaei S Mirzaei M. The lived experience of HIV-infected patients in the face of a positive diagnosis of the disease: a phenomenological study. AIDS Res Ther. (2021) 18:1–8. 10.1186/s12981-021-00421-4 34876162 PMC8650359

[8] Tefera E Mavhandu-Mudzusi AH. Experiences of antiretroviral therapy initiation among HIV-positive adults in Ethiopia: a descriptive phenomenological design. HIV/AIDS Res Palliative Care. (2022) 14:243–54. 10.2147/HIV.S361913 PMC9148206 35637644

[9] Patel RC Odoyo J Anand K Stanford-Moore G Wakhungu I Bukusi EA Facilitators and barriers of antiretroviral therapy initiation among HIV discordant couples in Kenya: qualitative insights from a pre-exposure prophylaxis implementation study. PLoS One. (2016) 11(12):e0168057. 10.1371/journal.pone.0168057 27930740 PMC5145201

[10] Franse CB Kayigamba FR Bakker MI Mugisha V Bagiruwigize E Mitchell KR Linkage to HIV care before and after the introduction of provider-initiated testing and counselling in six Rwandan health facilities. AIDS care. (2017) 29(3):326–34. 10.1080/09540121.2016.1220475 27539782

[11] Adams AK Zamberia AM. I will take ARVs once my body deteriorates”: an analysis of Swazi men’s perceptions and acceptability of test and start. Afr J AIDS Res. (2017) 16(4):295–303. 10.2989/16085906.2017.1362015 29132279

[12] Poku RA Owusu AY Mullen PD Markham C McCurdy SA. Antiretroviral therapy maintenance among HIV-positive women in Ghana: the influence of poverty. AIDS care. (2020) 32(6):779–84. 10.1080/09540121.2019.1653434 31405289

[13] Earnshaw VA Bogart LM Courtney I Zanoni H Bangsberg DR Orrell C Exploring treatment needs and expectations for people living with HIV in South Africa: a qualitative study. AIDS Behav. (2018) 22:2543–52. 10.1007/s10461-018-2101-x 29619585 PMC6051887

[14] Tefera E Mavhandu-Mudzusi AH. Experiences of Antiretroviral Therapy Initiation Among HIV-Positive Adults in Ethiopia: A Descriptive Phenomenological Design. Auckland: Dove Medical Press Ltd. (2022). p. 243–54. 10.2147/HIV.S361913 PMC9148206 35637644

[15] Seeley J Bond V Yang B Floyd S MacLeod D Viljoen L Understanding the time needed to link to care and start ART in seven HPTN 071 (PopART) study communities in Zambia and South Africa. AIDS Behav. (2019) 23:929–46. 10.1007/s10461-018-2335-7 30415432 PMC6458981

[16] Haacker M Birungi C. Poverty as a barrier to antiretroviral therapy access for people living with HIV/AIDS in Kenya. Afr J AIDS Res. (2018) 17(2):145–52. 10.2989/16085906.2018.1475401 30003850

[17] Kebaabetswe P Manyake K Kadima E Auletta-Young C Chakalisa U Sekoto T Barriers and facilitators to linkage to care and ART initiation in the setting of high ART coverage in Botswana. AIDS care. (2020) 32(6):722–8. 10.1080/09540121.2019.1640843 31298037 PMC6954979

[18] Manyazewal T. Using the world health organization health system building blocks through survey of healthcare professionals to determine the performance of public healthcare facilities. Arch Public Health. (2017) 75(1):1–8. 10.1186/s13690-017-0221-9 29075485 PMC5651704

[19] Pell C Reis R Dlamini N Moyer E Vernooij E. Then her neighbour will not know her status’: how health providers advocate antiretroviral therapy under universal test and treat. Int Health. (2019) 11(1):36–41. 10.1093/inthealth/ihy058 30137387

[20] Turan B Hatcher AM Weiser SD Johnson MO Rice WS Turan JM. Framing mechanisms linking HIV-related stigma, adherence to treatment, and health outcomes. Am J Public Health. (2017) 107(6):863–9. 10.2105/AJPH.2017.303744 28426316 PMC5425866

[21] Castro A. Adherence to antiretroviral therapy: merging the clinical and social course of AIDS. PLoS Med. (2005) 2(12):e338. 10.1371/journal.pmed.0020338 16187735 PMC1240055

[22] Ahmed A Dujaili JA Jabeen M Umair MM Chuah L-H Hashmi FK Barriers and enablers for adherence to antiretroviral therapy among people living with HIV/AIDS in the era of COVID-19: a qualitative study from Pakistan. Front Pharmacol. (2022) 12:3968. 10.3389/fphar.2021.807446 PMC8832364 35153763

[23] Wasti SP Simkhada P Randall J Freeman JV van Teijlingen E. Factors influencing adherence to antiretroviral treatment in Nepal: a mixed-methods study. PLoS One. (2012) 7(5):e35547. 10.1371/journal.pone.0035547 22563464 PMC3341373

[24] Arnold EA Rebchook GM Kegeles SM. Triply cursed’: racism, homophobia and HIV-related stigma are barriers to regular HIV testing, treatment adherence and disclosure among young black gay men. Cult Health Sex. (2014) 16(6):710–22. 10.1080/13691058.2014.905706 24784224 PMC4061253

[25] Yehia BR Stewart L Momplaisir F Mody A Holtzman CW Jacobs LM Barriers and facilitators to patient retention in HIV care. BMC Infect Dis. (2015) 15(1):1–10. 10.1186/s12879-015-0990-0 26123158 PMC4485864

[26] Nam SL Fielding K Avalos A Dickinson D Gaolathe T Geissler PW. The relationship of acceptance or denial of HIV-status to antiretroviral adherence among adult HIV patients in urban Botswana. Soc Sci Med. (2008) 67(2):301–10. 10.1016/j.socscimed.2008.03.042 18455285

[27] Wang M Miller JD Collins SM Santoso MV Wekesa P Okochi H Social support mitigates negative impact of food insecurity on antiretroviral adherence among postpartum women in western Kenya. AIDS Behav. (2020) 24:2885–94. 10.1007/s10461-020-02839-9 32212069 PMC7483232

[28] Ammon N Mason S Corkery J. Factors impacting antiretroviral therapy adherence among human immunodeficiency virus–positive adolescents in Sub-Saharan Africa: a systematic review. Public Health. (2018) 157:20–31. 10.1016/j.puhe.2017.12.010 29501984

[29] Croome N Ahluwalia M Hughes LD Abas M. Patient-reported barriers and facilitators to antiretroviral adherence in sub-Saharan Africa. AIDS. (2017) 31(7):995. 10.1097/QAD.0000000000001416 28121707 PMC5378008

[30] Singh V Lata S. A systematic review of HIV/AIDS related stigma among children and youth living with HIV. IAHRW Int J Soc Sci Rev. (2018) 6(10):1961–7.

[31] Neville Miller A Rubin DL. Factors leading to self-disclosure of a positive HIV diagnosis in Nairobi, Kenya: people living with HIV/AIDS in the Sub-Sahara. Qual Health Res. (2007) 17(5):586–98. 10.1177/1049732307301498 17478642

[32] Qiu Y Luo D Cheng R Xiao Y Chen X Huang Z Emotional problems and related factors in patients with HIV/AIDS. Zhong nan da xue xue bao. Yi Xue Ban J Cent South Univ Medi Sci. (2014) 39(8):835–41. 10.3969/j.issn.1672-7347.2014.08.014 25202953

[33] Martawinarti RN Nursalam N Andri SW. Lived experience of people living with HIV/AIDS undergoing antiretroviral therapy: a qualitative study. Jurnal Ners. (2020) 15(2):157163. 10.20473/jn.v15i1Sp.19002

[34] Areri H Marshall A Harvey G. Exploring self-management of adults living with hiv on antiretroviral therapy in north-west Ethiopia: qualitative study. HIV/AIDS Res Palliative Care. (2020) 20:809–20. 10.2147/HIV.S287562 PMC7725142 33312002

[35] Solomon N Molla M Ketema B. “I want to perform and succeed more than those who are HIV-seronegative” lived experiences of youth who acquired HIV perinetally and attend Zewditu Memorial hospital ART clinic, Addis Ababa, Ethiopia. PLoS One. (2021) 16(5):e0251848. 10.1371/journal.pone.0251848 34043659 PMC8158987

[36] Palella F Jr Armon C Chmiel JS Brooks JT Hart R Lichtenstein K CD4 Cell count at initiation of ART, long-term likelihood of achieving CD4 > 750 cells/mm3 and mortality risk. J Antimicrob Chemother. (2016) 71(9):2654–62. 10.1093/jac/dkw196 27330061

[37] Holtz CS Sowell R Velasquez G. Oaxacan women with HIV/AIDS: resiliency in the face of poverty, stigma, and social isolation. Women Health. (2012) 52(6):517–35. 10.1080/03630242.2012.690839 22860701

[38] Dibb B. Assessing stigma, disclosure regret and posttraumatic growth in people living with HIV. AIDS Behav. (2018) 22(12):3916–23. 10.1007/s10461-018-2230-2 30030741 PMC6208894

[39] Kelbert EF Pinheiro LM Souza LDM Pinheiro CAT Pinheiro KAT Motta JVS Suicide risk in people living with AIDS/HIV: the effect of childhood trauma is mediated by mental disorders and social vulnerability. AIDS care. (2020) 32(4):512–7. 10.1080/09540121.2019.1695732 31801367

[40] MacLean JR Wetherall K. The association between HIV-stigma and depressive symptoms among people living with HIV/AIDS: a systematic review of studies conducted in South Africa. J Affect Disord. (2021) 287:125–37. 10.1016/j.jad.2021.03.027 33780828

[41] Yabes JM Schnarrs PW Foster LB Scott PT Okulicz JF Hakre S. The 3 levels of HIV stigma in the United States military: perspectives from service members living with HIV. BMC Public Health. (2021) 21:1–11. 10.1186/s12889-021-11462-9 34266390 PMC8281656

[42] Ballouz T Gebara N Rizk N. HIV-related stigma among health-care workers in the MENA region. Lancet HIV. (2020) 7(5):e311–3. 10.1016/S2352-3018(19)30401-1 31928930

[43] Stephens JH Surjan A. BARRIERS Preventing access by men who have sex with men to HIV-related health services in Southeast Asia: a scoping review. Glob Public Health. (2022) 17(2):235–53. 10.1080/17441692.2020.1858922 33317394

[44] Hedge B Devan K Catalan J Cheshire A Ridge D. HIV-related stigma in the UK then and now: to what extent are we on track to eliminate stigma? A qualitative investigation. BMC Public Health. (2021) 21(1):1–10. 10.1186/s12889-021-11000-7 34053441 PMC8166014

[45] Setlhare V Wright A Couper I. The experiences of people living with HIV/AIDS in Gaborone, Botswana: stigma, its consequences and coping mechanisms. S Afr Fam Pract. (2014) 56(6):309–13. 10.1080/20786190.2014.975484

[46] Yu C-H Huang C-Y Ko N-Y Tung H-H Huang H-M Cheng S-F. The lived experiences of stigmatization in the process of HIV status disclosure among people living with HIV in Taiwan. Int J Environ Res Public Health. (2021) 18(10):5089. 10.3390/ijerph18105089 34064970 PMC8150537

[47] Tshuma S. The challenges faced by adolescents with perinatal HIV/AIDS *(dissertation)*. University of Pretoria, Pretoria, South Africa (2015).

[48] Nuraidah N Wanda D Hayati H Rachmawati IN Waluyo A. I can live a normal life”: exploring adherence to antiretroviral therapy in Indonesian adolescents living with HIV. Belitung Nurs J. (2022) 8(2):108–14. 10.33546/bnj.2024 37521893 PMC10386811

